# 519 Congruence of clinical suspicion of invasive fungal wound infection and biopsy positivity in burn patients

**DOI:** 10.1093/jbcr/irac012.150

**Published:** 2022-03-23

**Authors:** Kaitlin A Pruskowski, Garrett W Britton, Thomas A Mitchell, Leopoldo C Cancio

**Affiliations:** US Army Institute of Surgical Research, JBSA Fort Sam Houston, Texas; US Army Institute of Surgical Research, JBSA Fort Sam Houston, Texas; US Army Institute of Surgical Research, JBSA Fort Sam Houston, Texas; US Army Institute of Surgical Research, JBSA Fort Sam Houston, Texas

## Abstract

**Introduction:**

Invasive fungal wound infection (FWI) in burn patients is a high-mortality complication; early diagnosis and treatment may improve outcomes. Management of suspected FWI includes initiating broad-spectrum antifungals, obtaining a biopsy for histopathology and culture, and performing urgent surgical excision. However, the relationship between clinical suspicion (manifested by initiation of antifungals) and histopathological diagnosis is unknown.

**Methods:**

Patients admitted between 2004 and 2019 to our burn center, and initiated on any systemic antifungal, were identified. The electronic medical record (EMR) was reviewed to determine the indication for such therapy. Patients were included if antifungals were initiated out of concern for FWI. If the indication was not clear, patients were included if the systemic antifungal agent(s) initiated were triazoles (not fluconazole), echinocandins, or amphotericin B.

**Results:**

Two hundred one patients who received 251 courses of broad-spectrum antifungal therapy were included. Thirty six patients (17.9%) received more than one course of antifungal therapy. One hundred sixty five (82%) patients were male, with an average age of 41.1 ± 17.7 years. The average burn size was 49.7 ± 22.8% total body surface area (TBSA) and 60 (29.8%) patients had inhalation injury. The median time from injury to antifungal initiation was 17.5 days (IQR 10.7, 38.6 days).

One hundred sixty eight biopsies were obtained within 3 days of antifungal initiation. Seventy five biopsies showed FWC (44.6% of biopsies), 37 had FWI (i.e. fungi were identified either in viable tissue or angioinvasion) (22% of biopsies), and 56 had negative biopsies (33.3% of biopsies). Despite presence of fungi on histopathology, there were only 112 positive fungal wound cultures (44.6% of 251 antifungal courses) within 3 days initiation of antifungal therapy. *Aspergillus* was the most commonly isolated genus (n=47) followed by *Candida* (n=46), *Fusarium* (n=26), *Mucor* (n=10), and other (n=20). There were 35 instances where multiple fungal organisms were recovered in tissue culture. One hundred five patients (52.2%) died during their hospital stay; 38 of these patients had FWC, 25 had FWI, 20 had negative biopsies, and 22 did not have biopsies taken.

**Conclusions:**

Of 251 systemic antifungal courses initiated out of concern for FWI, FWI was biopsy-proven 14.7% of the time. Antifungal stewardship is needed to better identify appropriate high-risk patients for FWI. The development of a novel criteria or scoring system may be warranted to assist in deciding when to initiate systemic antifungal therapy for FWI in burn patients.

